# A stabilization device significantly improves measurement reliability and efficiency of Graf ultrasonography for developmental dysplasia of the hip: a retrospective cohort study

**DOI:** 10.3389/fped.2026.1902579

**Published:** 2026-08-07

**Authors:** Yehui Lan, Liyu Tang, Miao Jin, Bin Du, Jianfei Yan, Quanzhou Wu, Chenying Lu, Yanhua Huang, Zhihui Chen

**Affiliations:** 1 Department of Ultrasound, Lishui Central Hospital, The Fifth Hospital Affiliated to Wenzhou Medical University, Lishui Hospital of Zhejiang University, Lishui, Zhejiang, China; 2 Department of Orthopedics, Lishui Central Hospital, The Fifth Hospital Affiliated to Wenzhou Medical University, Lishui Hospital of Zhejiang University, Lishui, Zhejiang, China; 3 Department of Radiology, Lishui Central Hospital, The Fifth Hospital Affiliated to Wenzhou Medical University, Lishui Hospital of Zhejiang University, Lishui, Zhejiang, China

**Keywords:** developmental dysplasia of the hip, Graf method, reliability, stabilization device, ultrasound

## Abstract

**Objectives:**

To compare the measurement reliability, procedural efficiency, and infant stress responses between device-assisted and manual restraint approaches in Graf hip ultrasonography for developmental dysplasia of the hip (DDH) screening.

**Methods:**

In this retrospective cohort study, 238 infants (aged 0–6 months) underwent consecutive hip ultrasound examinations using both a Graf stabilization device and manual restraint during the same visit. Each examination included triple measurements of *α* and *β* angles by a single experienced sonographer. Primary outcomes included angle measurements and Graf classification. Examination time and infant crying incidence were also recorded. Intra-observer reliability for repeated measurements within each technique and inter-technique agreement between the two methods were evaluated separately using intraclass correlation coefficients (ICC) and Cohen's kappa.

**Results:**

No significant differences were observed in *α* angle (67.00° vs. 67.50°, *P* = 0.313), *β* angle (71.00° vs. 69.00°, *P* = 0.179), or Graf classification distribution (*P* = 0.832) between device-assisted and manual methods. However, the device-assisted approach showed significantly shorter examination time (*P* < 0.001) and a 13.5-fold reduction in crying incidence (0.84% vs. 11.34%, *P* < 0.001). Intra-observer reliability was excellent with device assistance (*α* angle: ICC = 0.95, *β* angle: ICC = 0.90) but poor with manual restraint (*α* angle: ICC = 0.49, *β* angle: ICC = 0.23). Inter-technique agreement was moderate for *α* angle (ICC = 0.71) and substantial for Graf classification (*κ* = 0.66), but poor with *β* angle (ICC = 0.36).

**Conclusion:**

The use of a stabilization device in Graf hip ultrasonography significantly improves measurement reliability and may enhance examination efficiency and infant comfort without altering diagnostic outcomes, supporting its integration into high-volume DDH screening programs.

## 1 Introduction

Developmental dysplasia of the hip (DDH) is the most prevalent orthopedic disorder in infants, and timely diagnosis and treatment are crucial to prevent long-term complications and the need for surgical interventions (1–3). The standard screening protocol for DDH includes general physical examination and selective hip ultrasound assessment (4). Among various ultrasound techniques, the static Graf method, which quantifies the *α* angle and *β* angle on standardized coronal images of the hip joint surface, is the most commonly used approach (5, 6). Although widely adopted, this method has been criticized for its susceptibility to potential intra- and inter-operator variability (7). Previous studies have shown that over 50% of Graf examinations fail to meet standardized imaging criteria, primarily due to improper positioning, incorrect identification of anatomical landmarks, and angular measurement errors (5, 8). To address these issues, Graf proposed a dedicated stabilization device incorporating infant immobilization and probe guidance mechanisms (9, 10). While such devices are commonly used in Europe (11), their adoption remains limited in many other regions, including China, due to cost, spatial, and workflow constraints. Moreover, recent evidence indicates that even with specialized positioning equipment and standardized training, pediatricians still require 50–180 clinical examinations to attain stable diagnostic competence (12). Therefore, there is a critical need to evaluate whether this pragmatic, manual approach yields diagnostic outcomes comparable to device-assisted scans when performed by experienced operators.

Voitl et al. provided preliminary evidence suggesting that manual restraint might achieve image quality comparable to device-assisted scans for normal (Type I) hips (10). However, the clinical applicability of these findings is limited by several factors, including the exclusion of pathological hip subtypes, a lack of quantitative assessments of infant comfort, and the absence of within-patient comparisons. Therefore, this study aims to directly compare the Graf stabilization device with manual restraint by evaluating their diagnostic agreement, procedural efficiency, and infant stress responses across a broader spectrum of Graf hip types.

## 2 Materials and methods

### 2.1 Study design and population

This retrospective cohort study included infants who underwent routine ultrasound screening for DDH at our Hospital from December 2020 to July 2023. Ethical approval was obtained from the Research Ethics Committee of our hospital [approval number: 2025(I)-374-01], and written informed consent was obtained from parents or legal guardians before enrollment. All eligible infants were aged between 0 and 6 months and successfully underwent three device-assisted examinations followed by manual assessments during the same visit. Participants who had received previous treatment, presented with other developmental deformities (such as congenital multiple joint contractures) in addition to DDH, or exhibited pathological hip dislocation were excluded.

### 2.2 Ultrasonic examination

All examinations were performed by a single ultrasound physician with extensive experience in pediatric hip ultrasonography, using a high-frequency linear array transducer (eL18-4, Philips EPIQ7). Standardized imaging parameters were maintained throughout the study with depth 3–4 cm, focal zone positioned at the cartilage–bone interface, and tissue harmonic imaging enabled to optimize image characterization. The Graf method was strictly followed for image acquisition and measurement (9, 10). The collected images were saved for subsequent evaluation, and all necessary data were entered into an Excel spreadsheet. This file contained all primary parameters for image evaluation, such as *α*-angle, *β*-angle, and Graf classifications. Secondary parameters including participant number, group (device-assisted or manual restraint), sex, age in weeks, duration of the examination, and infant crying (yes/no) were also recorded.

### 2.3 Positioning techniques

**Figure 1:**
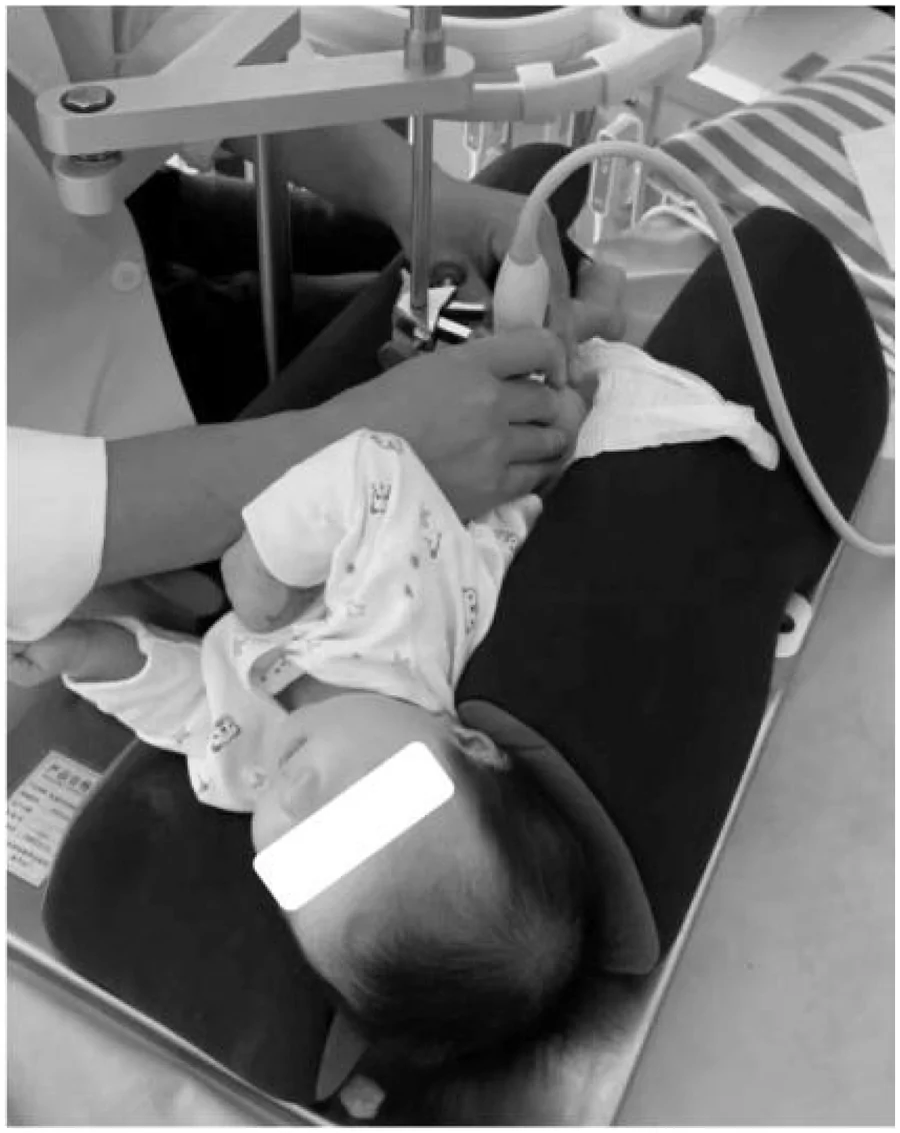
The stabilization device (combined with a positioning device and a probe guide system) as used in the device-assisted group (with baby in it).

All infants underwent two consecutive scanning sessions during the same session, with each protocol comprising triple measurements to ensure reliability. For the device-assisted examination, infants were positioned in lateral decubitus using the Graf stabilization system according to manufacturer guidelines (Figure 1) (9, 10). The device features an adjustable fixation unit with a longitudinal gap that accommodates infant placement. An adjustable sling was positioned across the device to support the infant while allowing hip accessibility. Tension was calibrated based on infant weight to achieve optimal positioning, ensuring slight hip protrusion beyond the device edges for transducer access. Lower limbs were maintained in natural flexion to prevent hip rotation, with knees positioned within the device confines to avoid scanning interference.

**Figure 2:**
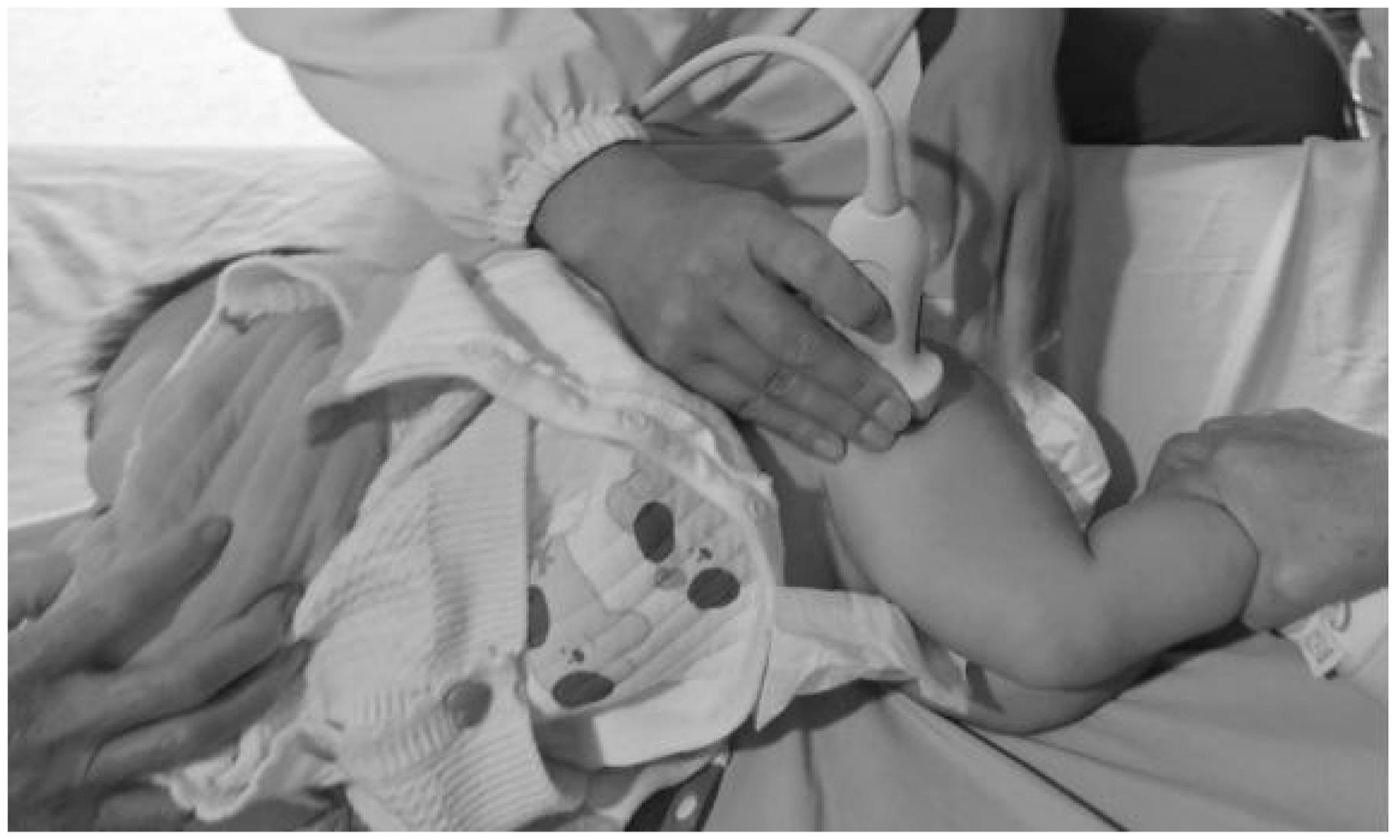
Manual fixation of a baby with hands as applied in the manual restraint group.

Following completion of the device-assisted examination with triple measurements, a traditional manual examination was immediately performed. Infants were repositioned on the standard examination couch and gently rotated to a lateral decubitus position. A parent or assistant provided manual stabilization by supporting the infant's torso and maintaining leg position to prevent rotation (Figure 2). The same triple-measurement protocol was followed during manual examination to maintain methodological consistency. The examination sequence (device-assisted followed by manual) remained consistent for all participants to eliminate order effects. Total examination time for each technique was recorded from initial transducer contact to completion of all three measurements. Infant comfort was assessed dichotomously (crying/not crying) during each examination phase by an independent observer. Prior to the commencement of each examination (both device-assisted and manual), the infant's state was confirmed to be calm. If the infant was crying, the procedure was paused and resumed only after the infant had settled and/or fallen asleep.

### 2.4 Image analysis and measurement protocol

**Figure 3:**
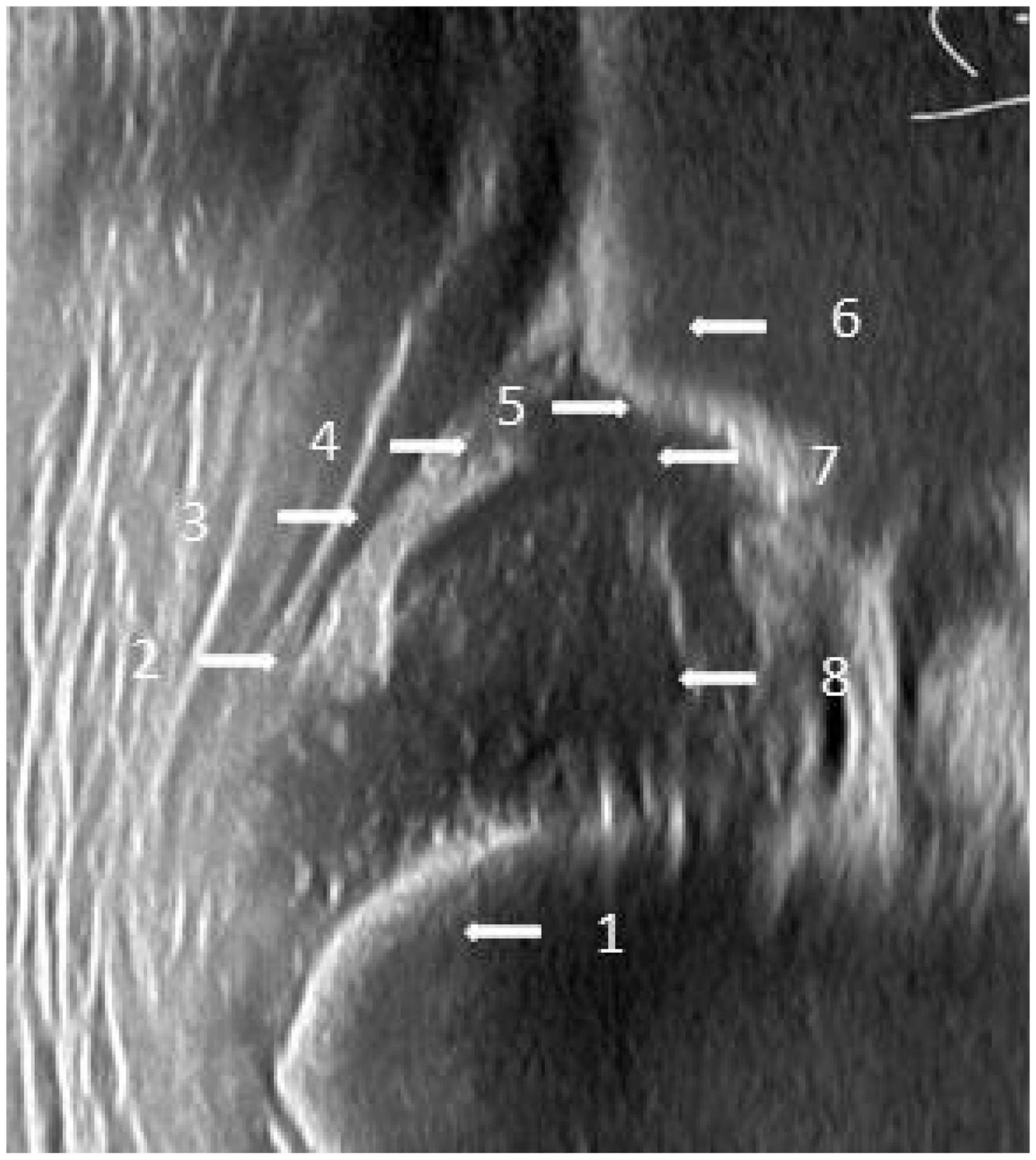
Anatomical landmarks: (1) chondro-osseous border, (2) synovial fold, (3) joint capsule, (4) labrum, (5) turning point (from concavity to convexity), (6) bony roof, (7) cartilaginous roof, and (8) femoral head.

**Figure 4:**
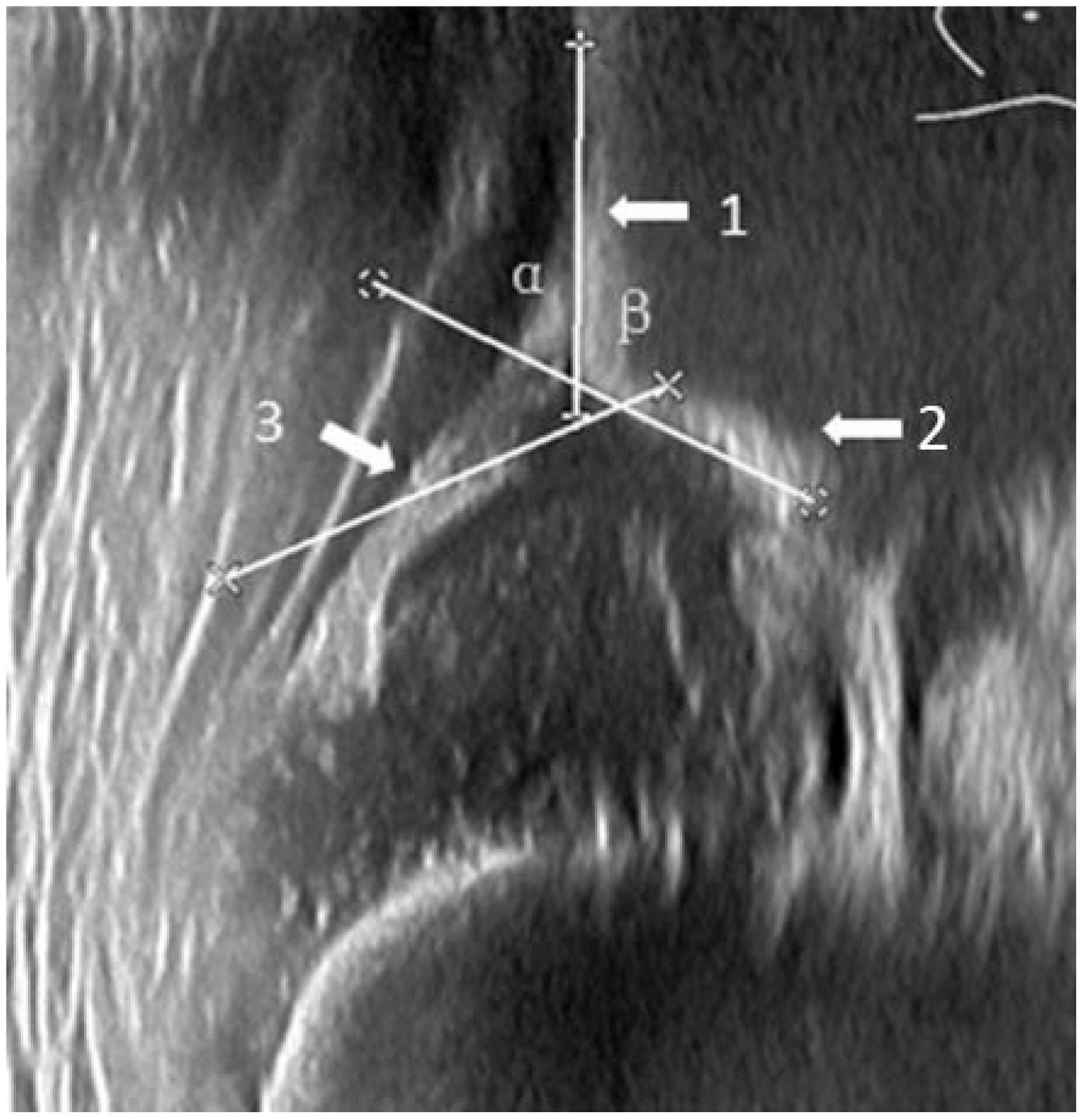
Measurement landmarks: (1) lower limb of ilium, (2) midsection of bony acetabular roof; (3) labrum.

**Figure 5:**
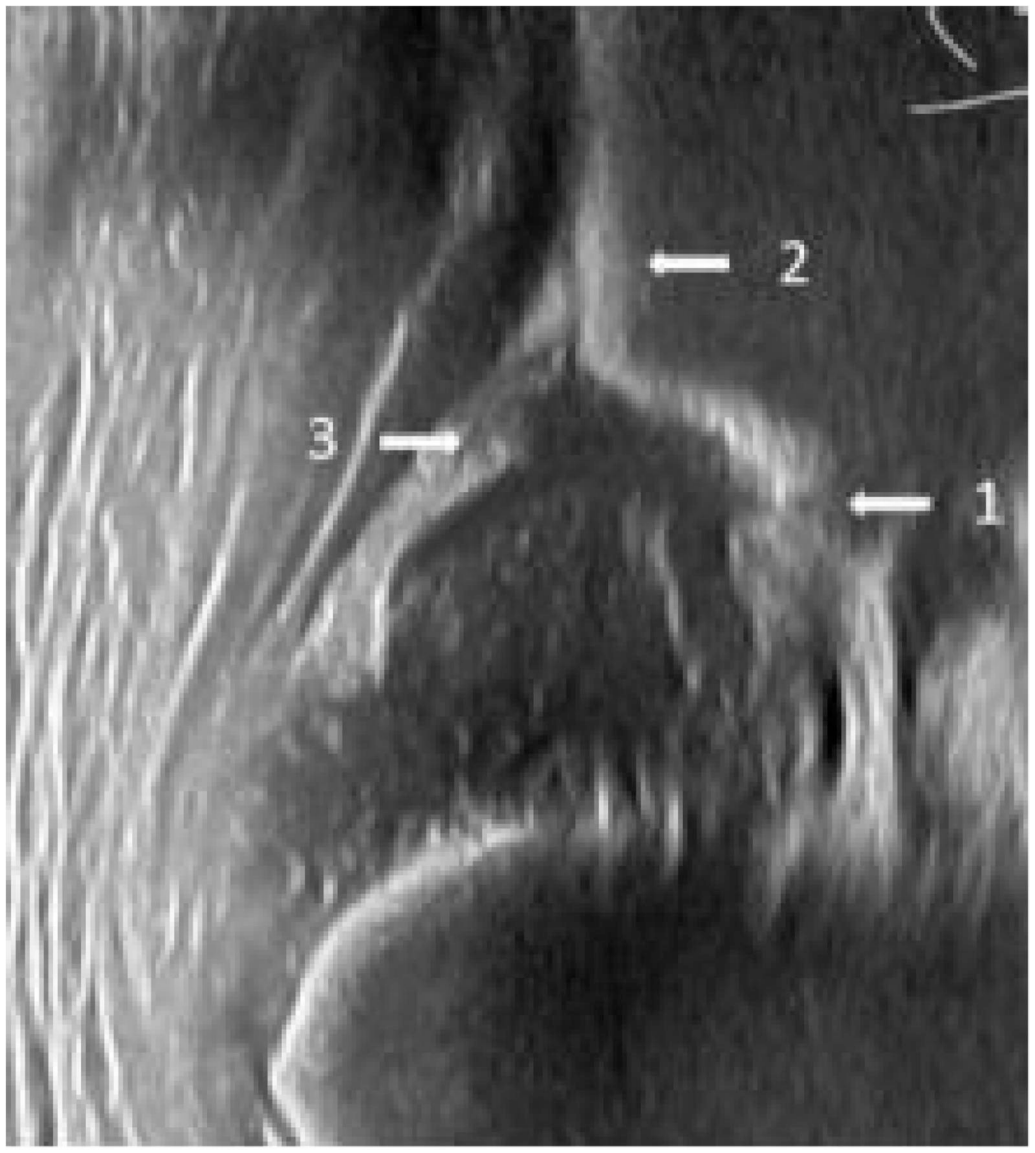
Three lines for angle measurement: (1) the baseline line, (2) the bone roof line, (3) the cartilage roof line, *α* and *β* angle.

All images were acquired according to standard Graf methodology (9, 13). In the standard coronal plane, eight essential anatomical landmarks were identified, including the chondro-osseous border, synovial fold, joint capsule, labrum, turning point (from concavity to convexity), bony roof, cartilaginous roof, and femoral head (Figure 3). After confirming the usability of the image for analysis, we further determined its suitability for measurement according to Professor Graf's requirements (13). This included assessing the lower limb of the ilium, the midsection of the bony acetabular roof, and the labrum (Figure 4). Subsequently, three lines were drawn for angle measurement: baseline, bony roof line, and cartilage roof line (Figure 5).

The measurement protocol adhered strictly to Graf specifications (8, 11). The *α* angle was measured between the iliac baseline and bony roof line, and the *β* angle between the iliac baseline and cartilaginous roof line. Based on the measurements, hips were classified as follows (9): Type I (*α* ≥ 60°), indicating a mature hip. Type IIa (*α* angle between 50° and 59°), observed in infants younger than 12 weeks and representing physiological immaturity, which was further subcategorized into IIa + (*α* angle meeting or exceeding the age-correlated minimum expected angle) and IIa− (*α* angle below the expected minimum, suggesting delayed ossification). Type IIb (*α* angle between 50° and 59°), found in infants aged 12 weeks or older, indicating delayed ossification. Type IIc (*α* angle between 43° and 49° with *β* < 77°) and Type D (*α* angle between 43° and 49° with *β* ≥ 77°). Type III (*α* < 43°, with an inverted labrum) and Type IV (*α* < 43°, without labral inversion), both representing dislocated hips. According to convention, hips classified as Type II or higher were considered developmentally immature. To ensure assessment completeness, hips classified as Graf Type III or Type IV (*n* = 11), which require specific management, were excluded from this study. Furthermore, no cases of Type IIc or Type D were identified. All measurements were performed by a single operator, with each qualified image measured three consecutive times for the analysis of immediate intra-observer variability.

### 2.5 Statistical analysis

The sample size of this retrospective study was determined by available clinical data accrued during the study period, and its adequacy is supported by precedent from the closest relevant investigation in this field (10), which found 117 infants were sufficient to detect meaningful differences between device-assisted and manual Graf ultrasonography. All statistical analyses were conducted using SPSS 25.0 software. The normality of continuous variables was assessed using Kolmogorov–Smirnov test, revealing that all quantitative variables in this study followed a non-normal distribution. Therefore, continuous data were presented as median with interquartile range (IQR), while categorical variables were shown as frequencies and percentages (%). Between-group comparisons (device-assisted vs. manual restraint) for non-normally distributed continuous variables, including *α* angle, *β* angle, and examination time, were conducted using the Mann–Whitney *U* test. The ordinal variable Graf classification was also compared between groups using this test. Differences in neonatal crying rates were analyzed using Fisher's exact test or chi-squared tests.

The reliability assessment framework followed established methodologies from prior hip ultrasonography studies, most notably the work of Chang et al. (14), where intraclass correlation coefficients (ICC) were used for continuous angle measurements, and Cohen's kappa was employed for categorical Graf classification to assess inter-observer agreement. Intra-observer reliability for the three consecutive measurements of *α* and *β* angles within each technique (device-assisted or manual) was evaluated using the ICC with a two-way mixed-effects model for absolute agreement. The agreement between the device-assisted and manual restraint techniques (inter-technique agreement) for *α* and *β* angles, and Graf classification was assessed using ICC and Cohen's kappa, respectively. Reliability values were interpreted as follows: <0.50, poor; 0.50–0.75, moderate; 0.75–0.90, good; and >0.90, excellent. A *P*-value <0.05 was considered statistically significant.

## 3 Results

### 3.1 Study population

The final analysis included 238 infants who successfully completed both examination protocols. As detailed in Table 1, the study population comprised 134 females (56.30%) and 104 males (43.70%). Age distribution analysis demonstrated that the majority of infants were in the 5–8 weeks age group (156 infants, 65.55%), followed by >12 weeks (30 infants, 12.61%), 9–12 weeks (28 infants, 11.76%), and ≤4 weeks (24 infants, 10.08%). This distribution reflects the typical screening window for DDH in our clinical practice.

**Table 1:** Baseline characteristics of the study cohort.

Characteristics	Infants, *n* (%)
Sex	
Female	134 (56.30)
Male	104 (43.70)
Age	
≤4 weeks	24 (10.08)
5–8 weeks	156 (65.55)
9–12 weeks	28 (11.76)
>12 weeks	30 (12.61)

DDH, developmental dysplasia of the hip.

### 3.2 Comparisons of outcome measurements between the device-assisted and manual restraint groups

Comprehensive comparisons of ultrasound measurements between the two examination techniques revealed no statistically significant differences (Table 2). No significant differences were found in *α* angle (device-assisted: 67.00° [IQR: 63.00–69.00] vs. manual: 67.50° [65.00–69.00], *P* = 0.313), *β* angle (71.00° [67.00–76.00] vs. 69.00° [67.00–76.00], *P* = 0.179), or Graf classification (*P* = 0.832). However, from the perspective of procedural outcomes, the device-assisted group demonstrated significantly shorter examination times compared to the manual restraint group (*P* < 0.001). Notably, device assistance was associated with a dramatic reduction in infant crying incidence (0.84% vs. 11.34%, *P* < 0.001).

**Table 2:** Comparisons of the ultrasound Graf screening results between the device-assisted group and the manual restraint group.

Variables	Device-assisted group (*n* = 238)	Manual restraint group (*n* = 238)	*P*-value
US parameters			
* α* angle (°)	67.00 (63.00, 69.00)	67.50 (65.00, 69.00)	0.313
* β* angle (°)	71.00 (67.00, 76.00)	69.00 (67.00, 76.00)	0.179
Graf classifications, *n* (%)			0.832
* *I	218 (91.60)	217 (91.18)	
* *IIa+	12 (5.04)	15 (6.30)	
* *IIa−	4 (1.68)	2 (0.84)	
* *IIb	4 (1.68)	4 (1.68)	
Examination time (s)	56.00 (49.00, 66.00)	77.00 (66.00, 89.75)	<0.001
Infant crying proportion, *n* (%)	2 (0.84)	27 (11.34)	<0.001

### 3.3 Intra-observer reliability and inter-technique agreement

Table 3 presents the inter-technique agreement between the device-assisted and manual restraint methods. Moderate inter-technique agreement was observed for *α*-angle measurements (ICC = 0.71, 95% CI: 0.65–0.77), while *β*-angle measurements showed poor inter-technique agreement (ICC = 0.36, 95% CI: 0.24–0.46). Graf classification demonstrated substantial inter-technique agreement (*κ* = 0.66, 95% CI: 0.50–0.83), indicating consistent diagnostic categorization regardless of examination method.

**Table 3:** Inter-group diagnostic agreement between the device-assisted group and the manual restraint group.

	ICC or Cohen's *κ* value	95%CI
US parameters		
*α* angle (°)	0.71	0.65–0.77
*β* angle (°)	0.36	0.24–0.46
Graf type	0.66	0.50–0.83

ICC, intraclass correlation coefficient; CI, confidence interval.

As detailed in Table 4, intra-observer reliability was superior with the device-assisted technique. For *α*-angle measurements, the device-assisted group showed excellent intra-observer reliability (ICC = 0.95, 95% CI: 0.93–0.96) compared to poor intra-observer reliability in the manual restraint group (ICC = 0.49, 95% CI: 0.42–0.56). Similarly, *β*-angle measurements revealed excellent reliability with device assistance (ICC = 0.90, 95% CI: 0.87–0.92) vs. poor reliability with manual restraint (ICC = 0.23, 95% CI: 0.15–0.31).

**Table 4:** Intra-group diagnostic agreement among infants with the Graf device or those with manual restraint.

	ICC	95% CI
Infants with the Graf device		
*α* angle (°)	0.95	0.93–0.96
*β* angle (°)	0.9	0.87–0.92
Infants with manual restraint		
*α* angle (°)	0.49	0.42–0.56
*β* angle (°)	0.23	0.15–0.31

ICC, intraclass correlation coefficient; CI, confidence interval.

## 4 Discussion

### 4.1 Main findings

In this retrospective cohort study, we systematically evaluated the diagnostic agreement, examination efficiency, and stress response in newborns undergoing Graf hip ultrasonography with a standardized stabilization device compared to those with conventional manual restraint. We also assessed intra-observer reliability for each technique. Our findings demonstrated that while the device-assisted method yielded comparable *α*-angle, *β*-angle, and final Graf classification relative to manual restraint, it significantly enhanced measurement reproducibility, reduced scanning time, and improved infant comfort.

### 4.2 Interpretations

The comparable *α* and *β* angles obtained by both techniques indicate that well-trained sonographers can acquire accurate Graf ultrasound images without a stabilization device. This reinforces the critical importance of adhering to the standardized anatomical plane (10, 13, 15). However, even minor deviations in probe alignment during freehand scanning can considerably alter the sonographic plane and introduce measurement variability (5, 8, 15). For example, Sioutis et al. reported that a transducer tilt of only 10° could change the *α* angle by approximately 8.7°, resulting in altered Graf classification in a great number of neonates (8). Consistent with this, our study revealed excellent intra-observer reliability for both *α* angle and *β* angles when using the stabilization device, in contrast to the poor reliability observed with manual restraint. This difference likely stems from the device's ability to maintain consistent probe perpendicularity, thereby reducing operator-dependent variability and ensuring highly reproducible measurements across repeated scans.

The enhanced reliability of *β* angle measurements with device assistance deserves special emphasis. Unlike the *α* angle, which is derived from clearly defined bony structures, the *β* angle reflects cartilaginous roof development and has consistently demonstrated higher inter-observer variability due to the subjective challenge of accurately identifying the labral tip (16, 17). Our findings suggest that this variability may be primarily attributed to technical factors, such as probe positioning, rather than being purely interpretive in nature. By standardizing image acquisition, the stabilization device effectively minimizes the technical variability, thus providing a more reliable evaluation of the cartilaginous roof for consistent grading of borderline hips. Beyond reliability, the substantial agreement in final Graf classification between the two methods is clinically reassuring. It confirms that, when performed by an experienced operator, both techniques support consistent diagnostic and management decisions for most hips. This aligns with previous reports affirming the robustness of Graf classification when the standard imaging plane is properly obtained (18, 19).

The device-assisted technique also offered significant practical advantages in efficiency and patient comfort. The streamlined, reproducible positioning minimized the need for probe adjustments, substantially reducing examination time, a key benefit for high-volume screening programs (10, 13). Furthermore, the 13.5-fold reduction in infant crying signifies substantially improved patient tolerance. The device's secure containment likely prevents the distress caused by prolonged manual restraint, thereby reducing movement artifacts and facilitating a quicker, higher-quality examination. This enhancement is critical for both workflow efficiency and parental satisfaction.

### 4.3 Strengths and limitations

This study possesses several strengths. To our knowledge, this is only the second study to directly compare device-assisted and manual restraint Graf ultrasonography. It significantly expands upon the preliminary work of Voitl et al. by including a broader range of Graf types (I, IIa+, IIa−, IIb) and implementing within-patient comparisons (10). Our study also incorporates quantitative assessments of procedural efficiency and infant comfort, which were absent in the prior research. The use of triple measurements for each method enhances the robustness of reliability analyses. Furthermore, all scans being performed by a single highly experienced sonographer minimizes inter-operator variability, allowing for a focused evaluation of the device effect itself.

However, several limitations should be acknowledged. The fixed examination sequence (device always first) may have introduced a sequence bias. The significantly higher crying incidence during the subsequent manual restraint could reflect cumulative infant irritability from the preceding scan, a factor noted by Graf regarding examination tolerance. Therefore, the observed differences in infant comfort (and potentially examination time) should be interpreted cautiously as secondary findings. The exclusion of Graf type III and IV hips, due to the inability to obtain the complete triple-measurement set, limits the generalizability of our findings to the most severe pathological subtypes. While the use of a single expert sonographer strengthens internal validity, it may limit the extrapolation of results to settings with less experienced operators. Future studies involving multiple sonographers with varying experience levels, employing a randomized examination sequence, and including adapted protocols for severe cases (Graf III/IV) are warranted to further validate and generalize these findings.

## 5 Conclusion

In conclusion, our findings indicate that utilizing a stabilization device for Graf ultrasonography significantly improves the examination process, primarily by enhancing measurement reliability, and potentially by reducing scanning time and increasing infant comfort without altering diagnostic outcomes. Based on these advantages, the integration of such devices into clinical practice should be considered, particularly in resource-limited or high-volume screening settings where standardized acquisition is challenging.

## Data Availability

The raw data supporting the conclusions of this article will be made available by the authors, without undue reservation.
